# Utilization of complementary and alternative medicine for the prevention of COVID-19 infection in Ghana: A national cross-sectional online survey

**DOI:** 10.1016/j.pmedr.2021.101633

**Published:** 2021-11-09

**Authors:** Irene A. Kretchy, Joseph A. Boadu, James-Paul Kretchy, Kofi Agyabeng, Alfred A. Passah, Augustina Koduah, Kwabena F.M. Opuni

**Affiliations:** a Department of Pharmacy Practice and Clinical Pharmacy, School of Pharmacy, University of Ghana, P.O. Box LG43, Legon, Accra, Ghana; b Department of Pharmaceutical Chemistry, School of Pharmacy, University of Ghana, P.O. Box LG43, Legon, Accra, Ghana; c Department of Physician Assistantship Studies, School of Medicine and Health Sciences, Central University, Miotso, Accra, Ghana; d Department of Mathematics, KU Leuven, Leuven, Belgium

**Keywords:** CAM use, Coronavirus, COVID-19, Herbs, Perception, Side effects

## Abstract

Complementary and alternative medicine (CAM) use is widespread and has played critical roles in preventing infections, including previous coronaviruses. This study sought to document current practices in the use of CAM for the prevention of COVID-19 disease in Ghana. An anonymous electronic survey was conducted from February 1, 2021 to April 30, 2021. Data on demographic characteristics, basic clinical information, illness perceptions about COVID-19, and CAM use during the pandemic period were generated. While about 82.5% (986/1195) of the participants used CAM during the COVID-19 period, 69.1% (681/986) of CAM users intented it for COVID-19 infection prevention. Vitamin supplements (88.1%, 869/986), spiritual healing/prayer (23.3%, 230/986), mineral supplements (22.3%, 220/986), botanical/herbal medicines (22.2%, 219/986), and diet therapy (19.4%, 191/986) were the main types of CAM used. From the adjusted binary logistic regression model, current age (aOR: 1.03, 95%CI: 1.01–1.05), sex (aOR: 1.41, 95%CI: 1.02–1.95), participants’ perceptions of consequences (aOR: 1.10, 95%CI: 1.04–1.17), identity (aOR: 1.15, 95%CI: 1.06–1.25) and concerns about COVID-19 (aOR: 0.91, 95%CI: 0.85–0.97) were statistically significant predictors of CAM use. These results suggest the need for appropriate public health policy on COVID-19 and CAM use in addition to directing further research initiatives toward an optimized COVID-19 prevention scheme using clinically validated CAM treatments. Research to validate the clinical efficacy of these products, especially the herbs, for COVID-19 prevention while isolating lead compounds that could be optimized and used for the treatment and prevention of COVID-19 is also recommended.

## Highlights

• 82.5% of all participants reported CAM use during the COVID-19 pandemic period.
• 69.1% of CAM users intended it for COVID-19 infection prevention.
• Commonly used CAM include vitamins, spiritual healing/prayer, minerals and herbals.
• Age, sex and COVID-19 illness perception significantly predicted CAM use.

## 1 Introduction

The Corona Virus Disease (COVID-19) which is a zoonotic viral infectious disease caused by severe acute respiratory syndrome coronavirus 2 has become a global pandemic of major public health concern (Gorbalenya et al., 2020). Worldwide, over 200 million cases and more than 4 million COVID-19 related deaths have been reported (COVID-19 Dashboard by the Center for Systems Science and Engineering (CSSE), 2021) with individuals having compromised immune systems, chronic diseases, and the elderly as high-risk populations (Wu and McGoogan, 2020). Although the World Health Organization developed preventive guidelines to slow viral spread (Wu and McGoogan, 2020), Medicine Regulatory Agencies in several countries have approved vaccines under emergency use authorisation and medicines like azithromycin, hydroxychloroquine, chloroquine phosphate, ivermectin, doxycycline, dexamethasone, methylprednisolone, remdesivir, and convalescent plasma for COVID-19 case management (Tarighi et al., 2021, Sanders et al., 2020).

With these conventional interventions, there are reports of complementary or alternative treatments such as herbal preparations, dietary therapy, vitamin supplements, and prayer for prevention or treatment of COVID-19 (Jabaris and Ananthalakshmi, 2021, Boozari and Hosseinzadeh, 2020, Panyod et al., 2020).

CAM involves medications, treatments, and medicinal practices used concurrently with (complementary) or in place of (alternative) conventional treatments (Ernst and Fugh-Berman, 2002, Lichtenstein and Waalen, 2002). Utilization of CAM in several countries is high (Harris et al., 2012, Kretchy et al., 2014). A review reporting CAM use in Sub-Saharan Africa ranged from 4.6% to 94% with an average of 58.2% (James et al., 2018). High availability, easy access, low costs of CAM (Kretchy et al., 2014, James et al., 2018, Debas et al., 2006), and perception towards illness (Canaway and Manderson, 2013, Kucuk, 2017) mainly contribute to its expanded utilization.

How people perceive illness is key to how they adopt particular health behaviours for prevention or management (Figueiras and Neto, 2019). Illness perceptions are usually cognitive or emotional representations of a disease that are predictive of coping (Broadbent et al., 2006) and based on the common-sense self-regulatory theory of illness representations (Leventhal et al., 1980). This theory proposes that people create mental representations of illness experiences through cognitive, and affective processes based on available information or symptom experiences (Broadbent et al., 2006). The theory proposes five components of cognitive illness perceptions, namely consequences, timelines, personal control, treatment control, and identity, while the emotional illness perception is based on concerns and negative emotional arousal of the illness (Broadbent et al., 2006, Leventhal et al., 2016).

Illness perception has successfully predicted behaviours and outcomes across populations including hypertension, diabetes, coronary heart disease, anxiety, depression, cancer and medication adherence (Foxwell et al., 2013, Broadbent et al., 2015, Alyami et al., 2021, Anakwa et al., 2021). In recent times, studies have explored illness perception and adherence to safety protocols for COVID-19 and observed that perceptions toward COVID-19 had significant impact on adherence to these measures (Chong et al., 2020). The physical and psychological impact of illness perception during the COVID-19 pandemic as well as the role of CAM and integrative therapies as coping health behaviours in response to perceptions about COVID-19 have also been reported (Kristoffersen et al., Man et al., 2020, Chong et al., 2021, Skapinakis et al., 2020, Dias Neto et al., 2021).

Natural medicines have played essential roles in previous coronavirus infection prevention (Boozari and Hosseinzadeh, 2020) and threats of the current COVID-19 with its associated risk perceptions has led to CAM utilization as well (Paudyal et al., 2021). Herbs such as *Allium sativum*; *Camellia sinensis*; *Zingiber officinale*, *Nigella sativa*, *Glycyrrhiza glabra*, and *Astragalus membranaceus* were used for preventing previous coronavirus infections (Luo et al., 2020) with vitamin and mineral supplements reported for COVID-19 prevention (Gasmi et al., 2021, Kretchy et al., 2021).

CAM use in the treatment and management of diseases is prevalent (Kretchy et al., 2014, Kretchy et al., 2021, Kuunibe and Domanban, 2012, Yarney et al., 2013), yet information on COVID-19 perception influencing CAM is limited. This study therefore aimed to assess the role of illness perceptions towards COVID-19 and CAM utilization while documenting the prevalence and pattern of use. Understanding these relationships will inform further research initiatives toward optimized COVID-19 prevention using clinically validated CAM treatments.

## 2 Methods

The study was approved by the Institutional Review Board of the Noguchi Memorial Institute for Medical Research, University of Ghana (CPN: 028/20-21) and conducted in accordance with the Helsinki Declaration. Participants were voluntarily recruited after they had been informed about the purpose of the study. All study participants approved online written informed consent before commencement of data collection.

### 2.1 Study design and participants

This was a national cross-sectional online study using anonymous electronic survey from February to April 2021 via an online platform (https://ee.kobotoolbox.org/single/2q6zUPlK). The survey tool was circulated through emails, professional association groups, and five social media platforms – Telegram, WhatsApp, Instagram, LinkedIn, and Facebook. Due to the threatening second wave of COVID-19 and risks associated with conducting face-to-face community-based national surveys, the data collection process was online, and participants were recruited across all sixteen regions of Ghana using a mix of convenience and snowball sampling approaches to increase the number of participants.

Participants were Ghanaian residents aged 18 years or more, able to understand the questions in English, and had access to internet. Screening questions to ascertain age and residency status of participants were used.

Participation was voluntary after brief information on study objectives, confidentiality, and estimated completion time were provided. Participants had access to the link after informed consent was obtained. Participants were requested to share the invitation with their contacts. To control for multiple submissions, the survey settings were set to reject multiple responses from the same IP address.

### 2.2 Data collection

A 3-paged 30-item questionnaire was used to generate data on demographic characteristics, basic clinical information, illness perceptions about COVID-19, and CAM use during the pandemic (See Fig. 2 for complete list of CAM therapies). Demographic and clinical questions included sex, age, region of residence, educational status, existing health condition and COVID-19 risk vulnerability. Questions on COVID-19 tests and outcomes were indicated. The frequency, type, patterns, sources, reasons, beliefs, and side effects of CAM use was also noted. Examples of questions asked included *(1) Which of the following CAM have you used during the COVID-19 pandemic period? (2) Was the CAM intended to prevent COVID-19 infection? (3) How long have you been using the CAM during the COVID-19 pandemic? and (4) Where did you receive the information on CAM?* Participants’ perceptions about COVID-19 were assessed with the 9-item Brief Illness Perception Questionnaire (Broadbent et al., 2006). The items are scored on a 10-point Likert scale (except for item 9) for consequences, timeline, personal control, treatment control, identity, concern, illness understanding, and emotional response dimensions to COVID-19 perceptions. Item 9, an open-ended question on beliefs about causes of illness was excluded from this study, and in line with previous research, the overall score for the B-IPQ and Cronbach’s alpha were not computed because each subscale is measured by only one item (Anakwa et al., 2021, Haines et al., 2019).

To minimize non-response and high drop-out rates, the questionnaire was kept as short as possible, and participants could answer all questions within 5 min. The adaptive questioning approach was also applied to minimize the response time of participants by conditionally showing specific questions based on responses to previous items.

An online pre-test of the questionnaire was conducted among 20 people who were not members of the research team and played no role in the design of the questionnaire, to ensure that the electronic version was user-friendly, technically functional, and comprehensible by prospective participants. Prior to piloting the online version, face-to-face interviews with consideration for strict adherence to the COVID-19 prevention protocols were conducted among 7 people to ensure that the questions were comprehensible and coherent.

### 2.3 Data analysis

Data collected through the electronic questionnaire were extracted into excel for cross-validation and cleaning and exported to STATA version 15 (StataCorp LLC, USA) for analysis. The cross-validation involved checking consistencies in responses and ensuring that all respondents met the inclusion criteria through the use of frequency and cross-tabulation of responses. Descriptive statistics of responses for categorical variables were reported with frequencies and percentages while that of continuous variables were reported in terms of means and standard deviations when normally distributed but reported as median with interquartile ranges when skewed. Bar charts were used to graphically display the distribution of existing health conditions of study participants, CAM used during the COVID-19 pandemic period, sources of information on CAM and sources of CAM among the study participants. Pearson Moment Correlation Coefficient was used in assessing the relationship between the various sub domains of perceptions about COVID-19 illness. A binary logistic regression model was used to assess the effects of background characteristics and illness perceptions about COVID-19 on the use of CAM. The results of the model were reported as odds ratios with their 95% confidence intervals. All statistical tests of significance were conducted at a 5% level.

## 3 Results

### 3.1 Background characteristics of study participants

**Fig. 1:**
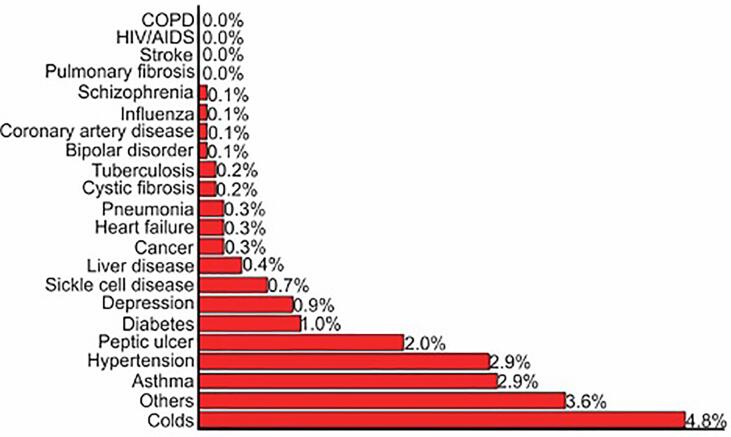
Existing health conditions of study participants.

This study had 1,195 participants in total. Participants had a median age of 25 years with more than two-thirds of them between ages 18 and 30 years inclusive. Few (2.6%, 31/1195) participants were 51 years and beyond. The majority (53.9%, 644/1195) were females. Almost all (93.5%, 1117/1195) participants were Christians and had tertiary level education (94.5%, 1129/1195). Less than a third (332/1195) of the participants had tested for COVID-19 out of which 17.5% (58/332) were positive (Table 1). Three common existing health conditions among participants were common cold (4.8%, 57/1195), asthma (2.9%, 35/1195), and hypertension (2.9%, 34/1195) (Fig. 1).

**Table 1:** Background characteristics of study participants.

	**Frequency**	**Percent**
**Current Age**		
Median (LQ, UQ)	25 (21, 33)	
18–30	837	70.0
31–40	223	18.7
41–50	104	8.7
>=51	31	2.6
**Sex**		
Female	644	53.9
Male	551	46.1
**Religion**		
Christianity	1,117	93.5
Islam	69	5.8
Traditionalism	3	0.3
No religion	6	0.5
**Educational Level**		
Primary	6	0.5
Secondary	60	5.0
Tertiary	1,129	94.5
**Tested for COVID-19**		
No	863	72.2
Yes	332	27.8
**Test results**		
Negative	274	82.5
Positive	58	17.5

### 3.2 Pattern of CAM use among study participants

**Fig. 2:**
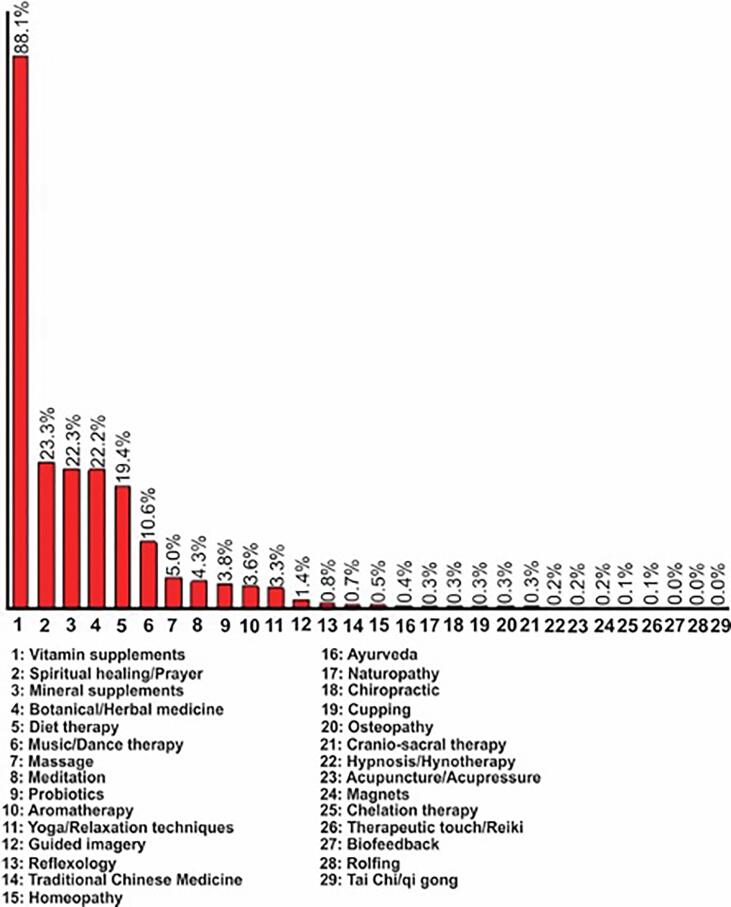
CAMs used during the COVID-19 pandemic period by study participants.

**Fig. 3:**
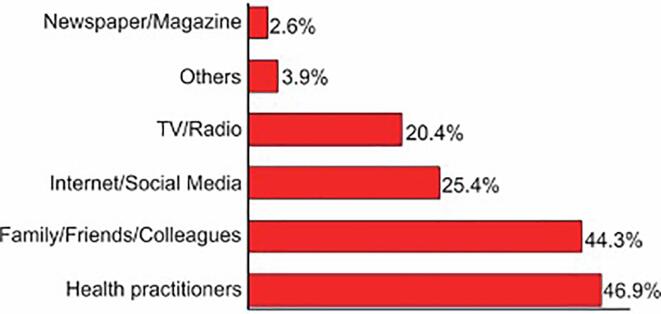
Source of Information on CAM.

In all 82.5% (986/1195) of the participants used some form of CAM during the COVID-19 period. Five commonly used CAM were vitamin supplements (88.1%, 869/986), spiritual healing/prayer (23.3%, 230/986), mineral supplements (22.3%, 220/986), botanical/herbal medicines (22.2%, 219/986), and diet therapy (19.4%, 191/986). The nature of CAM was mainly raw material/homemade (21.8%, 215/986) and finished products (75.3%, 742/986) (Table 2). The raw materials/homemade preparations were reported for use either individually or as combination remedies (see Appendix A). The least used CAM were magnets therapy, chelation therapy, and therapeutic touch/reiki (Fig. 2). About 86.5% (853/986) reported no side effects to CAM (Table 2). Participants’ source of information on CAM was mostly from health practitioners (46.9%, 462/1195) or Family/Friends/Colleagues (44.3%, 436/1195) (Fig. 3).

**Table 2:** Pattern of CAM use among study participants.

	**Frequency**	**Percent**
**CAM use**		
No	209	17.5
Yes	986	82.5
**Nature of the CAM**		
Raw materials/home-made	215	21.8
Finished product	742	75.3
Other (specify)	29	2.9
**CAM registered by the FDA**		
Yes	722	73.2
No	76	7.7
Don't Know	105	10.7
Not Applicable	83	8.4
**Duration for using the CAM during the COVID-19**		
<1 month	252	25.6
1–3 months	303	30.7
4–6 months	122	12.4
More than 6 months	309	31.3
**How did you decide on the frequency of therapy**		
Followed product label instructions	267	27.1
Followed practitioner’s instructions	224	22.7
Decided myself	478	48.5
Media (e.g. TV, Radio, Newspapers)	99	10.0
Friends/Relatives	157	15.9
Social media	63	6.4
Internet	83	8.4
Other	10	1.0
**CAM intended to prevent COVID-19 infection**		
Yes	681	69.1
No	305	30.9
**How often did you use the CAMs**		
Daily	574	58.2
Weekly	242	24.6
Monthly	88	8.9
Annually	13	1.3
Biannually	2	0.2
Other	67	6.8
**CAMs are effective in preventing COVID-19 infections**		
Strongly Agree	182	18.5
Agree	439	44.5
Neutral	304	30.8
Disagree	44	4.5
Strongly Disagree	17	1.7
**Safe to use CAM in preventing COVID-19 infection**		
Strongly Agree	148	15.0
Agree	478	48.5
Neutral	296	30.0
Disagree	55	5.6
Strongly Disagree	9	0.9
**Side effects experienced**^a)^		
Headache	33	3.35
Dizziness	25	2.54
Diarrhoea	23	2.33
Fatigue	21	2.13
Nausea	20	2.03
Allergic reactions	17	1.72
Vomiting	10	1.01
Pain	7	0.71
Bleeding	1	0.1
Bruising skin	1	0.1
Dermatitis	1	0.1

^a)^Multiple responses.

**Fig. 4:**
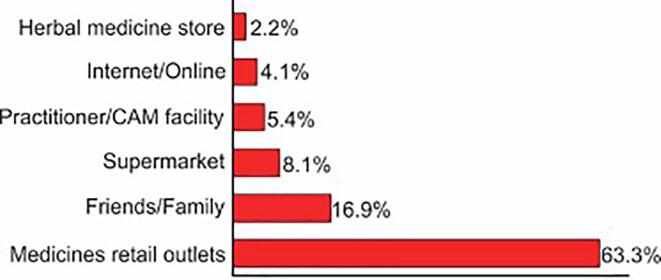
Sources of CAM among the study participants.

Medicines retail outlets (pharmacy and chemical shops) were the major sources of CAM acquisition while Herbal stores were the least (Fig. 4). The CAMs used were mostly finished products (75.3%, 742/986) and were registered by the Food and Drugs Authority of Ghana (73.2%, 722/986). About a third of participants had used CAM for more than six months (31.3%, 309/986) and 69.1% (681/986) of CAM use during this period being intended for COVID-19 infection prevention. More than half (58.2%, 574/986) of the participants took CAM daily (Table 2). The frequency of use was mostly based on personal decisions (48.5%, 478/986) with a few from social media. Similar proportions of CAM users agreed or strongly agreed to CAM being effective (63%, 621/986) and safe (63.5%, 626/986) for use in COVID-19 infection prevention (Table 2).

### 3.3 Perceptions about COVID-19 among study participants

COVID-19 illness representations included consequences of the infection (4.61 + 2.96), timeliness of living with the illness (5.23 + 2.93), personal control over the virus (4.84 + 2.78), identity with experience of COVID-19 symptoms (1.65 + 2.30) and concerns about COVID-19 (7.45 + 2.80) (Table 3).

**Table 3:** Perceptions about COVID-19 among study participants.

		**Pearson Moment Correlation Coefficient**							
	**Mean + SD**	**Consequence**	**Timeline**	**Personal control**	**Treatment control**	**Identity**	**Concern**	**Understanding**	**Emotional response**
Consequence	4.61 + 2.96	1							
Timeline	5.23 + 2.93	0.34*	1						
Personal control	4.84 + 2.78	0.04	−0.04	1					
Treatment control	4.41 + 2.35	−0.06*	−0.08*	0.28*	1				
Identity	1.65 + 2.30	0.16*	0.13*	−0.05	−0.07*	1			
Concern	7.45 + 2.80	0.21*	0.18*	0.01	−0.13*	−0.01	1		
Understanding	2.25 + 2.42	−0.06*	−0.12*	0.18*	0.17*	0.05	−0.27*	1	
Emotional response	4.62 + 2.98	0.34*	0.17*	0.08*	−0.05	0.18*	0.33*	−0.03	1

SD: Standard Deviation, *P-value < 0.05.

### 3.4 Effects of background characteristics and perceptions about COVID-19 on the use of CAM

From the binary logistic regression model, current age, sex, participants’ perceptions on consequence, identity, and concern about COVID-19 were the only statistically significant predictors of CAM use (Table 4).

**Table 4:** Effects of background characteristics and perceptions about COVID-19 on the use of CAM.

	**Unadjusted Binary Logistic Regression Model**				**Adjusted Binary Logistic Regression Model**		
	**uOR**	**95%CI**	**P-value**		**aOR**	**95%CI**	**P-value**
**Current Age**	1.04	1.02–1.06	<0.001***		1.03	1.01–1.05	0.005**
**Sex**			0.190				0.036*
Male	1.00				1.00		
Female	1.22	0.91–1.65			1.41	1.02–1.95	
**Religion**			0.680				0.602
Christian	1.13	0.63–2.03			1.18	0.64–2.18	
other	1.00				1.00		
**Educational Level**			0.250				0.706
Non-tertiary	1.00				1.00		
Tertiary	1.42	0.78–2.57			1.13	0.6–2.15	
**Existing Health Conditions**			<0.001***				0.064
No	1.00				1.00		
Yes	0.46	0.28–0.75			0.62	0.37–1.03	
**Perceptions about COVID-19**							
Consequence	1.14	1.08–1.2	<0.001***		1.10	1.04–1.17	0.001**
Timeline	1.07	1.02–1.13	0.010*		1.02	0.96–1.07	0.580
Personal control	0.97	0.92–1.03	0.320		0.99	0.93–1.05	0.711
Treatment control	0.95	0.89–1.01	0.130		0.96	0.9–1.03	0.299
Identity	1.21	1.11–1.31	<0.001***		1.15	1.06–1.25	0.001**
Concern	0.97	0.92–1.03	0.300		0.91	0.85–0.97	0.003**
Understanding	0.99	0.93–1.05	0.660		0.98	0.92–1.05	0.563
Emotional response	1.09	1.04–1.15	<0.001***		1.05	0.99–1.12	0.079

*p-value < 0.05, **p-value < 0.01, ***p-value < 0.001, CI: Confidence interval, aOR: Adjusted odds ratio, uOR: unadjusted odds ratio.

The odds of using CAM increased by 3% for each year increase in age (aOR: 1.03, 95%CI: 1.01–1.05). Female participants had 41% higher odds of using CAM compared with their male counterparts (aOR: 1.41, 95%CI: 1.02–1.95).

Each unit increase in participants’ perception of the consequences of COVID-19 led to a 10% increase in the odds of using CAM (aOR: 1.10, 95%CI: 1.04–1.17). A unit increase in beliefs about COVID-19 symptom experience was associated with a 15% increase in the odds of using CAM (aOR: 1.15, 95%CI: 1.06–1.25). The odds of using CAM reduced by 9% with every unit decrease in participants’ level of concern about COVID-19 (aOR: 0.91, 95%CI: 0.85–0.97).

## 4 Discussion

This study investigated uptake of CAM for prevention of COVID-19 infection in Ghana to understand the trend and suggest context-specific alternatives to the management of the disease. A preponderance of our study participants were females, a finding that corresponded to national statistics where females formed a majority of 51.2% with higher life expectancy of 65 years, compared to 63 years in males (2010 Population Housing Census: National Analytical Report, 2021). The majority of our study participants had attained tertiary education and this is common with on-line studies where people who are literates can respond to such surveys (Hossain et al., 2020, Nekliudov et al., 2020). The study also recorded highest number of participants as Christians and this corroborates the report that about 70% of the Ghanaian population is estimated to be Christians (2010 Population Housing Census: National Analytical Report, 2021). Ghana and other Sub-Saharan African countries, the evidence of using faith-based approaches for health problems in combination with CAM has been reported (Gyasi et al., 2015, Okoronkwo et al., 2014).

In this study, less than a third of participants had tested for COVID-19 of which 17.5% were positive. This low rate of testing for COVID-19 confirms the low national trend in which approximately seven tests per 100, 000 people were recorded daily (Ghana’s Outbreak Response Management Updates, 2021). This portrays deficiencies in existing testing facilities and the lack of capacity to accommodate testing needs of the population of about 30 million, should there be a sudden increase in COVID-19 cases in the country. It is hoped that the government would improve testing capacity, by adequately equipping laboratories, since early detection of cases impact disease management and prevents spread.

Our findings also showed that common cold, asthma, and hypertension were common health conditions reported. It is known that whilst common cold/flu and fever may be experienced in the early stages of COVID-19, among other respiratory symptoms like cough and dyspnea (Ahmad et al., 2021), asthma, diabetes, and hypertension may complicate the outcome of coronavirus infections (Yawson et al., 2020).

The products used by the respondents were consistent with most frequently used CAM products in literature (Kretchy et al., 2021, Okoronkwo et al., 2014, Gyasi et al., 2015, Egede et al., 2002). While about 69.1% of CAM use was intended for the prevention of COVID-19 disease, they were generally considered effective and safe for the anticipated purpose. Vitamins were the most frequently used to prevent COVID-19 followed by spiritual healing/prayers, use of mineral supplements, and botanical/herbal medicines. High use of CAM products has been linked with medical conditions considered as life-threatening (Egede et al., 2002). Since CAM was reported to be useful in improving immunity in individuals, it has been suggested for use as COVID-19 prophylaxis. Traditional Chinese Medicine and Ayurveda have been used for COVID-19 infection prevention and/or treatment in countries like China and India (National Health Commission of the People’s Republic of China, 2021, Protocol, 2021). While dietary therapy and herbal medicines have also been suggested as complementary preventive therapy for COVID-19 (Panyod et al., 2020), a survey conducted in Iran revealed that the participants used herbal products, traditional medicines, and vitamin supplements to prevent them from contracting the virus (Erfani et al., 2020). Some of the herbs used as homemade CAM intervention in this study are in line with previous reports (Paudyal et al., 2021, Luo et al., 2020). The use of spiritual therapy is similar to other studies, which reported that prayer intervention was one of the common forms of CAM used among residents in Ghana and South African (Gyasi et al., 2015, Singh et al., 2004). Prayers are highly favoured within most African communities and may account for the high numbers of respondents resorting to this form of CAM (Okoronkwo et al., 2014, Gyasi et al., 2015, Singh et al., 2004). Most respondents sourced their CAM from medicines retail outlets (pharmacies and over-the-counter medicine sellers), and this is supported by previous work, in which the pharmacy was an important source for CAM (Gyasi et al., 2015). The medicine retail outlets are usually the first point of call for medical care (Okai et al., 2019). The majority of the respondents used CAM products because of personal decisions. Having a strong personal belief has been documented to play a critical role in individuals’ use of CAM (Gyasi et al., 2016). Based on the participants’ reports, and in line with a previous study on the use of herbal medicinal products (Kretchy et al., 2021), the majority of the CAM products were registered by the national Food and Drugs Authority (FDA), confirming a rise in the number of registered products by FDA Ghana from 2011 to 2019 (Kretchy et al., 2021) though not specific for COVID-19.

The demographic characteristics that significantly influenced the trend of CAM use were the age and sex of the participants. The findings indicated that the use of CAM during the COVID-19 increased with the increasing age of participants. Although previous studies on age and CAM use have reported mixed results, a review found that CAM use significantly increased with increasing age in 26 studies out of the 134 that were analysed (Bishop and Lewith, 2010). In relation to the findings on sex, this study observed that female participants had a higher chance (41%) of using CAM than males during the COVID-19 pandemic. Similar results have been documented for females being more likely to use CAM than males (Egede et al., 2002, Alwhaibi et al., 2015, Laiyemo et al., 2015, Alwhaibi and Sambamoorthi, 2016), which are congruent with our findings. Plausible reasons for this observation are that women are more motivated to use CAM because they tend to adopt more preventive healthcare approaches, as well as have more health needs that may not be sufficiently satisfied by conventional healthcare systems (Zhang et al., 2015, Kristoffersen et al., 2014).

The perceptions of people about COVID-19 will be relevant to how they prevent or adjust to the illness. These cognitive and emotional representations of COVID-19 have been reported to be formed at the initial stages of the pandemic and have remained quite stable or decreased over time (Dias Neto et al., 2021). In this study, participants formed their perceptions about COVID-19 based on both cognitive and emotional views about the disease which comprises the anticipated physical, emotional and social effects and outcomes of the illness on an individual’s wellbeing (Broadbent et al., 2006). Our study revealed that the participants’ perception of COVID-19 affecting their lives (consequences) led to the use of CAM. COVID-19 has resulted in the significant loss of many lives globally, an increase in unemployment rates, a reduction in food security as well as self-isolation of infected individuals (Piltch-Loeb et al., 2021, Aliakbari Dehkordi et al., 2020, VanderWeele, 2020, Yang et al., 2020, Mentis, 2021). These consequences are likely reasons for people’s use to CAM for COVID-19 prevention. Our study indicated that the beliefs about experiencing COVID-19 symptoms also resulted in a significant increase in CAM use. Some common symptoms of COVID-19 include fever, cough and dyspnoea (Pullen et al., 2020). However, there are more severe symptoms of COVID-19 such as breathlessness (from respiratory distress syndrome), sudden confusion, and constant chest pain (Valizadeh et al., 2020, Ahmad et al., 2020). The participants may have experienced any of these symptoms or want to avoid experiencing such symptoms and thus, resulting in their use of CAM in preventing COVID-19 infections. The results of this study also showed that the lower the concern about COVID-19, the lower the likelihood of using CAM. In the course of a pandemic, fear, worry, or even paranoia can easily spread throughout the population (Freckelton, 2020). This fear tends to push individuals to take drastic measures in avoiding infection. These measures include using readily available and affordable health interventions, such as herbal medicine and dietary supplements (Panyod et al., 2020, Freckelton, 2020, Alyami et al., 2020, Silveira et al., 2020). Thus, the lower the concerns, the less likely to take any action.

The strength of this study is in the use of an online survey that enabled the recruitment of study participants across all the regions of Ghana since the COVID-19 pandemic is a challenge to conducting face-to-face research. Also, this study provides comprehensive information on the prevalence, pattern, and perceptions towards CAM use for COVID-19 pandemic control in Ghana, which can serve as the basis for an improved COVID-19 management scheme using clinically validated CAM treatments.

There are however some limitations that need to be considered in the interpretation of the study findings. First, online data collection was used for this study and therefore only people who could read and write, have access to social media networks and access the internet could participate. Second, since convenience and snowball sampling were used in this study, the data may not be representative of the population distribution in Ghana in terms of age, sex, and place of residence. Hence, a disproportionately large number of study participants were female and had tertiary education, which is a common observation in online surveys (Hossain et al., 2020, Nekliudov et al., 2020). Third, the cross-sectional nature of this study prevents the analysis of causal relationships, and the use of self-reported measures introduces reporting bias. Finally, in considering the prevalence, pattern, and perceptions towards the COVID-19 pandemic and CAM use for its control, the long-term effects of CAM cannot be inferred from this study.

Despite these limitations, this study findings highlight some key clinical, research, and policy implications. The outcome of the study indicates a high prevalence of CAM use for COVID-19 prevention. Consequently, there is a clinical implication of potential herb-drug interaction for people who are on medications for chronic disease conditions. Thus, a rigorous health educational campaign is needed to avoid any potential public health crisis. In addition, a clear policy is needed for the use of CAM in the prevention and management of COVID-19. From this study, many people used different herbs for the prevention of COVID-19 infection and as a result, research could be conducted to validate the clinical efficacy of these herbs for COVID-19 prevention and possibly isolate lead compounds that could be optimized and used for the treatment and prevention of the coronavirus infection.

## 5 Conclusion

The study observed that the use of CAM for COVID-19 infection prevention was common with demographic and illness perceptions towards COVID-19, contributing to the use. The information could be used to educate the safe use of CAM while promoting research into the clinical and potential usefulness for the prevention and treatment of the COVID-19 infection.

## 6 Availability of data and materials

The data that support the findings of this study are available from the corresponding author, upon reasonable request.

## Funding

The project was self-funded by the authors.

### CRediT authorship contribution statement

**Irene A. Kretchy:** Conceptualization, Methodology, Formal analysis, Investigation, Writing – original draft, Writing – review & editing, Project administration. **Joseph A. Boadu:** Methodology, Investigation, Writing – original draft, Writing – review & editing. **James-Paul Kretchy:** Conceptualization, Investigation, Writing – original draft, Writing – review & editing, Project administration. **Kofi Agyabeng:** Methodology, Formal analysis, Writing – original draft, Writing – review & editing. **Alfred A. Passah:** Formal analysis, Writing – review & editing. **Augustina Koduah:** Methodology, Formal analysis, Investigation, Writing – review & editing. **Kwabena F.M. Opuni:** Conceptualization, Methodology, Formal analysis, Investigation, Writing – original draft, Writing – review & editing, Project administration.

## Declaration of Competing Interest

The authors declare that they have no known competing financial interests or personal relationships that could have appeared to influence the work reported in this paper.

## Figures and Tables

**Table A1:** Plants used as raw materials/homemade preparations for COVID-19 prevention.

	Common name	Scientific name
1	Clove	*Syzygium aromaticum*
2	Pineapple	*Ananas comosus*
3	Activated charcoal powder	n.a.
4	Dandelion	*Taraxacum officinale*
5	Cashew	*Anacardium occidentale*
6	Pawpaw	*Carica papaya*
7	Lemon	*Citrus Limonum*
8	Cinnamon	*Cinnamomum zeylanicum*
9	Lemongrass	*Cymbopogon citratus*
10	Ginger	*Zingiber officinale*
11	Chilli pepper	*Capsicum frutescens*
12	Neem	*Azadirachta indica*
13	Garlic	*Allium sativum*
14	Onion	*Allium cepa*
15	Negro pepper	*Xylopia aethiopica*
16	Grains of paradise	*Aframomum melegueta*
17	Cloves	*Syzygium aromaticum*
18	Hibiscus	*Hibiscus rosa-sinensis*
19	Moringa leaves	*Moringa oleifera*
20	Prekese	*Tetrapleura tetraptera*
21	Bitter root	*Lewisia rediviva*
22	Mango	*Mangifera indica*
23	African basil	*Ocimum gratissimum*
24	Pear	*Pyrus pyrifolia*
25	Turmeric	*Curcuma longa*
26	Black pepper	*Piper guineense*

n.a., not applicable
